# Glucagonoma-induced Dilated Cardiomyopathy in a Young Woman

**DOI:** 10.1210/jcemcr/luad008

**Published:** 2023-02-20

**Authors:** Jennifer Lourdes Ng, Lee Matthew Ponce, Gabriel Jasul

**Affiliations:** Section of Endocrinology, Diabetes and Metabolism, Department of Medicine St. Luke's Medical Center, Quezon City, Philippines 1112; Section of Endocrinology, Diabetes and Metabolism, Department of Medicine St. Luke's Medical Center, Quezon City, Philippines 1112; Section of Endocrinology, Diabetes and Metabolism, Department of Medicine St. Luke's Medical Center, Quezon City, Philippines 1112

**Keywords:** pancreatic neuroendocrine tumor, glucagonoma, dilated cardiomyopathy, thromboembolism, octreotide

## Abstract

A 27-year-old woman presented with an epigastric mass, accompanied by emesis and weight loss. An abdominal computed tomography (CT) scan showed a pancreatic head-body mass and liver metastasis. Biopsy revealed well-differentiated pancreatic neuroendocrine tumor, grade 3. Chromogranin and synaptophysin stains were positive, with a Ki-67 index of 33.6%. Increased frequency of episodic emesis with incomprehensible speech and right-sided weakness prompted admission. A cranial magnetic resonance imaging (MRI) scan showed a subacute cerebrovascular infarct. Serum glucagon was markedly elevated. On the third hospital day, neurologic symptoms progressed, with repeat cranial MRI demonstrating acute lacunar infarcts. Two-dimensional echocardiography was consistent with dilated cardiomyopathy. Because of her fragile condition, she was not a candidate for surgery and chemotherapy. She was treated with long-acting octreotide every 4 weeks for a total of 10 doses. Posttreatment, a 2-dimensional echocardiogram and an abdominal CT scan showed no significant change. We report a rare case of glucagonoma with associated prominently dilated cardiomyopathy and ischemic stroke.

Glucagonoma is a rare pancreatic neuroendocrine tumor with an incidence rate of 2.6 per 100 000 000 persons [1]. Associated manifestations of this condition include glossitis, diarrhea, weight loss, hyperglycemia, necrolytic migratory erythema, and anemia [1, 2]. Cardiomyopathy and cerebrovascular disease are unusual complications for these neoplasms and are even rarer in young people without other conditions. Here, we describe a case of a young woman with no known comorbidities who was eventually diagnosed with glucagonoma and developed dilated cardiomyopathy and ischemic stroke.

## Case Presentation

A 27-year-old Filipino woman with no known comorbidities was referred because of a 2-year history of a gradually enlarging abdominal mass. This was accompanied by nausea, nonbilious vomiting, abdominal pain, diarrhea, and undocumented weight loss. She also presented with intermittent oozing excoriations on both lower extremities. She had a family history of breast and liver malignancy, but there were no known premature cardiovascular diseases, coagulopathies, or endocrinopathies in the family. She denied smoking, alcoholic beverage intake, and use of illicit drugs.

**Figure 1.**
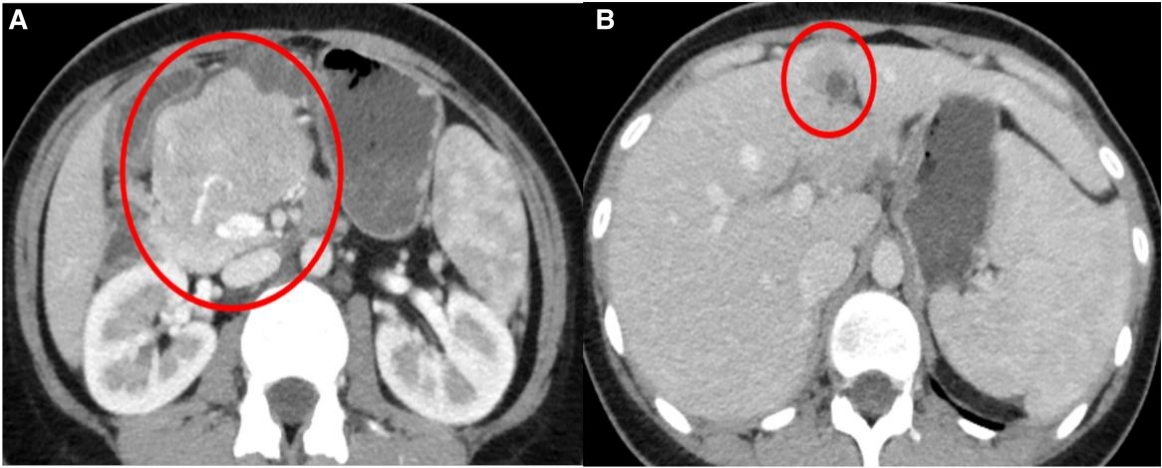
Contrast-enhanced axial computed tomography image of the abdomen showing a large, lobulated, enhancing soft-tissue mass arising from the pancreatic head-body with intralesional hyperdensities indicative of vascularity (A). An enhancing nodule with central fluid density at segment IVB of the liver suggestive of hepatic metastasis (B).

Abdominal computed tomography (CT) scan showed a heterogeneously enhancing, lobulated pancreatic head-body mass measuring 6.7 × 6.1 × 9.9 cm (Fig. 1). A hypo-enhancing nodule in segment IVb of the liver was also noted and was worrisome for metastasis. No lymphadenopathy or peritoneal carcinomatosis were seen.

**Figure 2.**
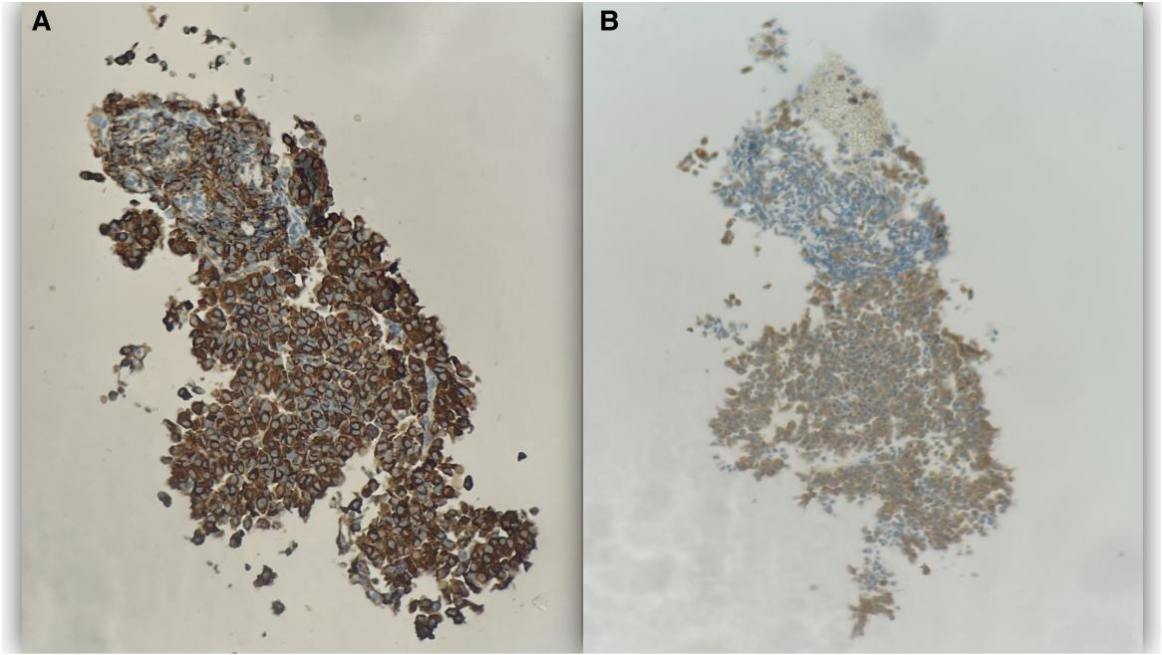
Positive staining with chromogranin (A) and synaptophysin (B).

Endoscopic-guided fine needle aspiration biopsy of the pancreatic mass was done. Immunohistochemical stains were negative for CA19-9 and SMAD-4, but positive for chromogranin and synaptophysin (Fig. 2). Ki-67 index was 33.6%. The lesion was classified as a well-differentiated pancreatic neuroendocrine tumor, grade 3.

**Figure 3.**
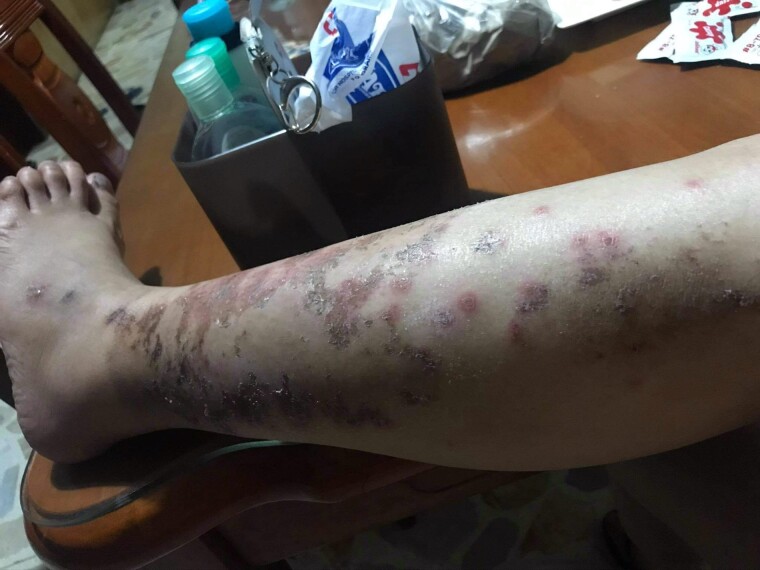
Multiple irregularly shaped, erythematous macules coalescing into patches with scales and crusting on the left leg.

She was initially lost to follow-up. After 4 months, she was admitted to the hospital for a 1-week history of increasingly frequent episodic emesis (up to 10 times per day) and abdominal discomfort, along with a 2-day history of incomprehensible speech and right-sided weakness. On physical examination, the patient was underweight (body mass index, 17.45 kg/m^2^). Her heart rate was elevated at 101 beats/min. Her skin was dry with hyperpigmented patches on both legs, which were consistent with the clinical picture of necrotizing migratory erythema (Fig. 3). She had pale conjunctivae and dry lips and oral mucosa. Jugular veins were flat. Heart sounds were distinct without any appreciable murmurs. There was a palpable, firm, nontender, nonmovable epigastric mass roughly measuring 10 × 7 cm. Pedal edema was absent. Neurologic examination showed a shallow right nasolabial fold with motor and sensory deficits of the right extremities.

Cranial magnetic resonance imaging (MRI) scans revealed an acute infarct in the left lentiform and caudate nuclei, insular cortex, and frontal coronal radiata. She was started on aspirin and atorvastatin. On the second hospital day, she started having diarrheal episodes, which were managed supportively.

Serologic workup for functional pancreatic neuroendocrine tumor showed the following: serum glucagon was markedly elevated (>25 000 pg/mL [>25 000 ng/L], normal <80), serum pancreatic polypeptide (6562 pg/mL [>6526 ng/L], normal <228), and gastrin (621 pg/mL [>621 ng/L], normal 13-115) were increased, and serum vasoactive intestinal peptide (<50 pg/mL [<50 ng/L], normal <75) was within normal limits. Other pertinent findings were prediabetes (fasting blood sugar, 112 mg/dL [6.216 mmol/L], hemoglobin A1c, 5.7%), normocytic, normochromic anemia (hemoglobin, 7.7 g/dL), and hypoalbuminemia (3.2 g/dL). Screening for MEN1 was unremarkable. The patient was referred to clinical nutritional support for nutritional assessment. No further workup for nutritional deficiencies were done because of limited funds.

**Figure 4.**
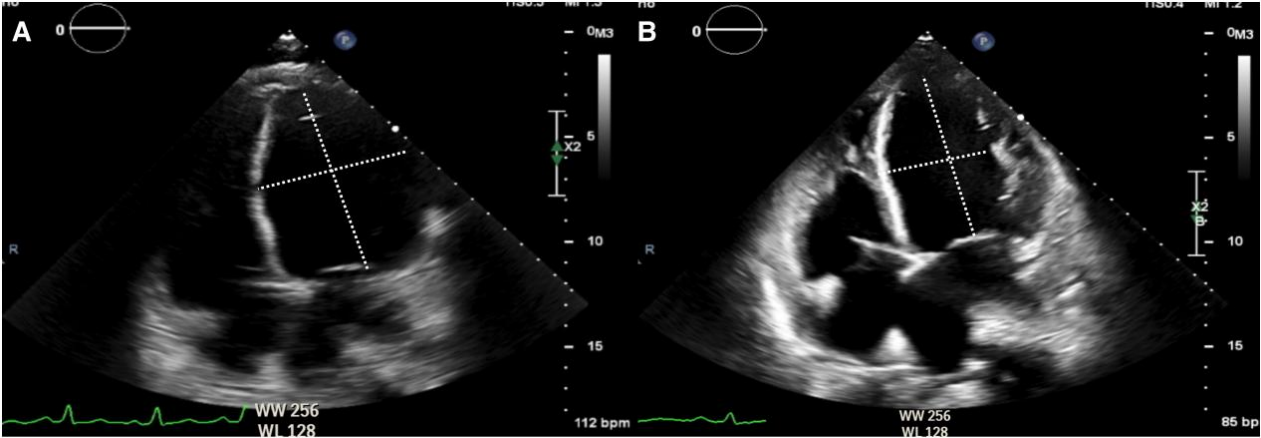
Two-dimensional echocardiography showing the apical 4-chamber view with the baseline echocardiography before treatment (A) and the repeat echocardiography after 10 sessions of octreotide long-acting repeatable (B). The left ventricular end diastolic volume was 6.0 cm and 6.4 cm before and after treatment, respectively (normal value, 3.9-5.3 cm for females).

On the third hospital day, her slurred speech and right-sided weakness progressed. A repeat cranial MRI scan revealed a new infarct in the precentral gyrus of the right frontal lobe. Aspirin was changed to enoxaparin. Workup for hypercoagulability was pursued. Two-dimensional echocardiography showed depressed ejection fraction (20.9%), a dilated left ventricle with normal relative wall thickness, and global hypokinesia of the left ventricular wall (Fig. 4). Twelve-lead electrocardiogram indicated sinus tachycardia (113 beats/min) with prolonged QT interval (0.51 seconds) and abnormal R wave progression. Carotid and vertebral duplex scans, 24-hour Holter monitoring, and lipid profile were unremarkable. There was no evidence of thrombosis in either lower extremity on venous duplex scan. Coronary angiography, cardiac MRI, and myocardial perfusion scans were not done because of the patient’s fragile medical condition. Ischemic and valvular heart diseases were considered less likely as causes of the cardiomyopathy. The low ejection fraction was more likely attributable to the glucagonoma.

## Treatment

Because of the patient's fragile condition, she was not a candidate for surgery and chemotherapy. Instead, she underwent therapy with octreotide long-acting repeatable (LAR) 30 mg IM every 4 weeks. As part of her heart failure regimen, initiation of sacubitril + valsartan was attempted but was eventually discontinued because of hypotension. She was started on captopril, spironolactone, and carvedilol. Oral nutrition supplementation was included as part of her nutritional support.

## Outcome and Follow-up

Ten doses of 30 mg octreotide LAR were given. This was based on the placebo-controlled, double-blind, prospective, randomized (PROMID) study, and no uptitrations were made [3]. After treatment, a repeat 2—dimensional echocardiogram showed minimal improvement in ejection fraction (27% from baseline of 20.9%) with a dilated left ventricle and global hypokinetic walls. A repeat abdominal CT scan revealed no significant changes in the size of the pancreatic and liver masses. A follow-up chest CT scan did not show any pulmonary nodules, masses, or enlarged lymph nodes. The administration of prescribed therapies was sometimes delayed because of financial constraints. She reported improvement in appetite. However, rashes on the lower extremities would flare occasionally because of the delays in dosing with octreotide. She had no of shortness of breath, orthopnea, palpitations, or edema. Activities such as walking and climbing stairs were reportedly better tolerated. Despite the treatment, her cardiac function did not improve; hence, she was still deemed a poor candidate for surgery and chemotherapy.

## Discussion

The manifestations of glucagonoma syndrome result from supraphysiologic levels of the hormone glucagon. Glucagon promotes gluconeogenesis and glycogenolysis, leading to elevated blood glucose levels. Amino acids are mobilized and protein degradation is increased. As a consequence, patients are at risk for malnutrition and severe nutritional deficiencies manifesting as glossitis, stomatitis, or cheilitis. Moreover, necrotizing migratory erythema (NME) is believed to result from hypoaminoacidemia and zinc deficiency [2].

There are few case reports on glucagonoma-induced dilated cardiomyopathy. Chang-Chretien et al described a 54-year-old woman with a 7.0-cm pancreatic mass with lymph node metastasis and elevated glucagon concentration at 1261 pg/mL (normal, 20-100 pg/mL). She initially presented with glossitis, angular cheilitis, necrotizing migratory erythema, resting tachycardia, and depressed ejection fraction (15%). Surgical resection of the mass led to the resolution of symptoms and normalization of glucagon (26 pg/mL) and ejection fraction (55%) [4]. Zhang et al discussed the case of a 51-year-old woman with MEN1 syndrome, including metastatic glucagonoma, who developed cardiogenic shock secondary to acute decompensated heart failure with severely depressed ejection fraction (10%) despite treatment with octreotide LAR 2 weeks before admission. Octreotide infusion resulted in improved left ventricular function and recovery of ejection fraction (45%) [5]. Furthermore, Demir et al reported a case of a 64-year-old man with a 3.5-cm pancreatic mass and high glucagon concentration of 97 pmol/L (normal, <50 pmol/L). He was initially admitted for acute pulmonary edema and left ventricular failure with depressed ejection fraction (30%). Surgery resulted in normalization of glucagon and ejection fraction (67%) [6]. In 2019, Barabas et al detailed a case of a 67-year-old woman with a 5 × 3 cm pancreatic mass with liver metastasis and markedly elevated glucagon concentration of >500 pmol/L (normal, 0-50 pmol/L). She experienced 2 years of NME, weight loss, palpitations, peripheral edema, and 4 years of left ventricular impairment with depressed ejection fraction (25%). Lanreotide and a heart failure regimen provided clinical and biochemical improvement; however, her left ventricular dysfunction persisted with ejection fraction just reaching 28% [7].

In contrast to our case, the symptoms of the patients in the previous case reports manifested during the 5th to 7th decade of life. Similarly, all of these patients had reduced ejection fractions ranging from 10% to 38% secondary to a nonischemic dilated cardiomyopathy as documented by an angiogram or cardiac MRI scan. In our patient's case however, because of her fragile condition as well as financial constraints, advanced cardiac workup was not pursued at this time. The patient's clinical profile and 2-dimensional echocardiogram findings clearly suggest that the dilated cardiomyopathy was induced by the glucagonoma.

Cardiomyopathy is unusual in glucagonoma, and the mechanism of action is still not well understood. One proposed mechanism is that glucagon causes an increase in myocardial cAMP, resulting in increased heart rate and cardiac output [8]. This elevation of cAMP facilitates binding of myocardial cAMP to cyclic nucleotide-gated channels, leading to increased inward current by sodium and potassium ions, which is a determinant of cardiac automaticity [9]. Moreover, elevation of myocardial cAMP levels can lead to increased spontaneous, rhythmic calcium release from the sarcoplasmic reticulum via the ryanodine receptors, thereby affecting the excitation-contraction coupling of myocardial muscle cells [9]. These mechanisms result in increased cardiac inotropy and chronotropy. Consequently, the chronic stimulation to the heart could have led to the development of cardiomyopathy. The variability of glucagon receptor expression and the cross-reactivity with other G protein receptors in the heart may explain the preferential effect of hyperglucagonemia on the left ventricle [7].

As mentioned, surgery in the previously reported cases resulted in significant improvement of left ventricular function. However, our patient's poor cardiac function and severe malnutrition made surgery and chemotherapy prohibitive. Hence, she underwent somatostatin analogue therapy with octreotide LAR because it has both antisecretory and antiproliferative effects. Previous studies noted a decrease in glucagon secretion and reduction of NME, glossitis, and diarrhea after treatment. In addition, the PROMID study revealed that octreotide LAR has a significantly longer median time to tumor progression compared with placebo [3]. With regard to the cardiomyopathy, only 1 of 2 case reports showed significant improvement in left ventricular function after octreotide infusion [6, 7]. Despite clinical improvement, there was no recovery of left ventricular function and pancreatic mass after 10 doses of octreotide LAR and standard heart failure regimen for our patient. Therefore, similar to what was demonstrated in the study of Barabas et al, an alternate cause of cardiomyopathy cannot be entirely ruled out in our patient and/or chronic exposure to excessive glucagon levels may have already produced irreversible myocardial damage [7].

Interestingly, our patient also manifested with ischemic stroke. Glucagonomas are associated with venous thromboembolic events as reported in 30% to 80% of cases [10]. Increased factor X production by pancreatic α cells may promote activation of the coagulation cascade, whereas zinc deficiency resulting from hypoalbuminemia from her malabsorptive state may have affected the fibrinolytic system [10]. These mechanisms contribute to the prothrombotic state in glucagonoma. However, initial workup with protein C and S, factor V Leiden, homocysteine, and carotid, vertebral, and venous duplex scans were negative, which cannot explain our patient's predicament. Malignancy gives rise to a hypercoagulable state because of the ability of tumor cells to activate the coagulation system, therefore increasing the risk for both venous and arterial thromboembolism. The tumor itself may have activated the procoagulant factors promoting thrombosis and stroke. Our patient will need further workup for stroke in the young. For now, to the best of our knowledge, this is the first case of glucagonoma with acute ischemic stroke, a rather peculiar presentation.

## Learning Points

- It is recommended to perform cardiovascular evaluation at the time of glucagonoma diagnosis to institute timely and appropriate management of possible cardiomyopathy and thromboembolism.
- Further studies regarding the pathophysiology of cardiomyopathy and thromboembolism in glucagonoma are needed to allow the development of alternative therapies.
- Communication among the multidisciplinary team is of utmost importance to provide the best patient care.

## Data Availability

Original data generated and analyzed for this case report are included in this published article.

## Abbreviations

CT: computed tomography
LAR: long-acting repeatable
MRI: magnetic resonance imaging
NME: necrotizing migratory erythema
